# Two populations of neurokinin 1 receptor-expressing projection neurons in lamina I of the rat spinal cord that differ in AMPA receptor subunit composition and density of excitatory synaptic input

**DOI:** 10.1016/j.neuroscience.2010.03.028

**Published:** 2010-06-02

**Authors:** E. Polgár, K.S. Al Ghamdi, A.J. Todd

**Affiliations:** Neuroscience and Molecular Pharmacology, Faculty of Biomedical and Life Sciences, West Medical Building, University Avenue, University of Glasgow, Glasgow G12 8QQ, UK

**Keywords:** dorsal horn, pain, NK1 receptor, CGRP, VGLUT2, glutamatergic synapse, AMPAr, AMPA, receptor, CGRP, calcitonin gene-related peptide, LPb, lateral parabrachial area, LTP, long-term potentiation, NK1r, neurokinin 1 receptor, pERK, phosphorylated extracellular signal-regulated kinases, TSA, tyramide signal amplification, VGLUT, vesicular glutamate transporter

## Abstract

Lamina I of the spinal cord contains many projection neurons that express the neurokinin 1 receptor (NK1r). It has been reported that these cells can undergo long-term potentiation (LTP), which may result from insertion of AMPA-type glutamate receptors (AMPArs) containing GluA1 or GluA4 subunits. We therefore investigated synaptic AMPAr expression on these cells with immunocytochemistry following antigen-retrieval. We also examined their density of glutamatergic input (by analysing AMPAr synaptic puncta and contacts from glutamatergic boutons), and phosphorylation of extracellular signal-regulated kinases (pERKs) following noxious stimulation. Our results indicate that there are two populations of NK1r-expressing projection neurons: large GluA4^+^/GluA1^−^ cells with a high density of glutamatergic input and small GluA1^+^/GluA4^−^ cells with a much lower input density. Results from pERK experiments suggested that the two groups may not differ in the types of noxious stimulus that activate them. Glutamatergic synapses on distal dendrites of the large cells were significantly longer than those on proximal dendrites, which presumably compensates for the greater attenuation of distally-generated excitatory postsynaptic currents (EPSCs). Both types of cell received contacts from peptidergic primary afferents, however, on the large cells these appeared to constitute over half of the glutamatergic synapses, and were often associated with elongated AMPAr puncta. This suggests that these afferents, which probably contain substance P, provide a powerful, secure synaptic input to large NK1r-expressing projection neurons. These results demonstrate the importance of GluA4-containing AMPArs in nociceptive transmission and raise the possibility that different forms of LTP in lamina I projection neurons may be related to differential expression of GluA1/GluA4.

Lamina I is innervated by nociceptive primary afferents (Light and Perl, 1979; Sugiura et al., 1986), many of which contain the neuropeptide substance P (Lawson et al., 1997). It contains a relatively high density of neurons that project to the brain (Willis and Coggeshall, 1991; Craig, 1995; Villanueva and Bernard, 1999), and ∼80% of these cells express the neurokinin 1 receptor (NK1r) on which substance P acts (Spike et al., 2003). NK1r-expressing dorsal horn neurons (including projection cells) are activated by noxious stimuli (Salter and Henry, 1991; Doyle and Hunt, 1999; Todd et al., 2002) and are necessary for the development of hyperalgesia in neuropathic and inflammatory pain states (Mantyh et al., 1997; Nichols et al., 1999). Lamina I projection neurons have been classified into three morphological types: fusiform, pyramidal and multipolar (Zhang et al., 1996; Zhang and Craig, 1997; Yu et al., 1999; Todd et al., 2002; Spike et al., 2003; Almarestani et al., 2007), and it has been suggested that morphology is related to function, with pyramidal cells responding selectively to innocuous thermal stimuli (Han et al., 1998). However, we have reported that pyramidal-shaped lamina I projection neurons in the rat frequently express the NK1r, can be densely innervated by substance P-containing (nociceptive) primary afferents, and can respond to noxious stimulation (Todd et al., 2002; Spike et al., 2003; Al-Khater and Todd, 2009).

Glutamate released by primary afferents and axons of excitatory interneurons acts on AMPA receptors (AMPArs), which mediate fast excitatory synaptic transmission (Yoshimura and Jessell, 1990) and play a major role in perception of both acute and chronic pain (Dickenson et al., 1997; Garry and Fleetwood-Walker, 2004). AMPArs are tetramers that are made from four subunits, GluA1-4 (also known as GluR1-4/GluRA-D). Synaptic AMPArs are not detected in fixed tissue, as protein cross-linking prevents access of antibodies to the synaptic cleft and postsynaptic density. However, they can be revealed with an antigen retrieval method based on pepsin treatment (Watanabe et al., 1998; Nagy et al., 2004a). There is strong evidence that the punctate staining seen with AMPAr subunit antibodies after pepsin treatment corresponds to postsynaptic receptors at glutamatergic synapses (Watanabe et al., 1998). The staining colocalizes with that for other post-synaptic density proteins such as NMDA receptors and PSD-95 (Nagy et al., 2004b; Yasaka et al., 2009), is associated with glutamatergic boutons (Nagy et al., 2004a), and is absent in tissue from mice in which the corresponding subunit has been deleted (Polgár et al., 2008b; Todd et al., 2009). In addition, electron microscopy reveals that immunostaining is located at postsynaptic densities (Nagy et al., 2004a).

Subunit composition determines the properties of AMPArs. For example, the presence of GluA1 or GluA4 (both of which have long C-terminal tails) is necessary for the activity-dependent insertion of AMPArs that underlies certain forms of long-term potentiation (LTP) (Bredt and Nicoll, 2003; Esteban et al., 2003). NK1r-expressing lamina I projection neurons can undergo LTP following dorsal root stimulation at C fibre strength (Ikeda et al., 2003, 2006), and we have found that in lamina I, GluA1- and GluA4-containing receptors are present in largely non-overlapping synaptic populations (corresponding to approximately two-thirds and a quarter of the glutamatergic synapses, respectively, Polgár et al., 2008b). The initial aim of this study was therefore to determine whether GluA1 or GluA4 were present at excitatory synapses on NK1r-expressing projection neurons in lamina I. Since our results showed that large and small neurons differed in AMPAr subunit expression, we looked for further evidence that these corresponded to different functional populations by examining their glutamatergic inputs, and also their extracellular signal-regulated kinase (ERK) phosphorylation in response to noxious stimulation. In addition, we found that large NK1r-expressing projection neurons possessed very elongated AMPAr puncta and we tested whether these corresponded to synapses formed by peptidergic primary afferents, which are known to innervate these cells (Todd et al., 2002).

## Experimental procedures

All experiments were approved by the Ethical Review Process Applications Panel of the University of Glasgow, and were performed in accordance with the European Community directive 86/609/EC and the UK Animals (Scientific Procedures) Act 1986.

### Retrograde tracing

Thirteen Adult Wistar rats (240–320 g; Harlan, Loughborough, UK) were anaesthetized with either ketamine and xylazine (73.3 and 7.3 mg/kg i.p., respectively, supplemented as necessary) or with isoflurane. They were placed in a stereotaxic frame and in those cases in which isoflurane was used, this was administered through a mask attached to the frame. Each of these rats received an injection of 50 nl of 4% Fluorogold (Fluorochrome Inc, Englewood, CO, USA) targeted on the left lateral parabrachial area (LPb), through a glass micropipette. After a 3–5 day survival period, they were re-anaesthetized with pentobarbitone (300 mg i.p.) and perfused through the heart with fixative that contained 4% freshly depolymerized formaldehyde. Brain and lumbar spinal cord sections were removed and fixed for at least 4 h at 4 °C. The brain was cryoprotected in 30% sucrose overnight and the region containing the injection site was cut into 100 μm thick frozen sections, which were mounted in anti-fade medium and viewed with epi-fluorescent illumination and an UV filter set. Tissue from lumbar spinal cord segments L2–L5 was cut horizontally into 60 μm thick sections with a vibrating microtome, and these were processed as described below.

Six of these animals had also received an additional injection of cholera toxin B subunit into either the caudal ventrolateral or dorsal regions of the medulla. In these cases, other spinal segments were used in one of two other studies (Al Ghamdi et al., 2009; Polgár et al., 2010).

The LPb was chosen as an injection target because we have found that the vast majority of lamina I projection neurons can be labelled following injections of Fluorogold into this region in the rat (Al Ghamdi et al., 2009). Specifically, ∼97% of the lamina I neurons that project to the thalamus, periaqueductal grey matter or nucleus of the solitary tract are also retrogradely labelled from the LPb (Spike et al., 2003; Al-Khater and Todd, 2009; Polgár et al., 2010).

### AMPArs on NK1r-immunoreactive lamina I projection neurons

Many antigens, including the NK1r, cannot be detected following the treatment with pepsin that is needed to reveal synaptic AMPArs. However, we have shown that this problem can be circumvented by immunostaining with a tyramide signal amplification (TSA) method prior to pepsin treatment (Nagy et al., 2004a; Todd et al., 2009). Horizontal sections from the lumbar enlargement of 10 of the rats that had received Fluorogold injections into the LPb were initially incubated for 3 days at 4 °C in rabbit antiserum against the NK1r (Sigma, Poole, UK; cat number S8305; 1:200,000), which was detected with a TSA kit (tetramethylrhodamine; PerkinElmer Life Sciences, Boston, MA, USA) according to the manufacturer's instructions. They were rinsed and treated with pepsin (Dako, Glostrup, Denmark; 0.5 mg/mL for 10 min at 37 °C) as described previously (Watanabe et al., 1998; Nagy et al., 2004a; Todd et al., 2009). The sections were then incubated for 3 days in a cocktail consisting of guinea-pig anti-Fluorogold (Protos Biotech, Corp., New York; 1:500), mouse monoclonal antibody against GluA2 (Millipore, Watford, UK; Clone 6C4; 1:300) and either rabbit anti-GluA1 (Millipore; cat number AB1504; 1:300) or rabbit anti-GluA4 (Millipore; cat number 06-308; 1:400). These were revealed by overnight incubation in species-specific secondary antibodies: donkey anti-rabbit IgG conjugated to Alexa 488 (Invitrogen, Paisley, UK; 1:500), donkey anti-mouse IgG conjugated to Cy5 (Jackson Immunoresearch, West Grove, PA; 1:100) and biotinylated donkey anti-guinea pig IgG (Jackson Immunoresearch; 1:500). The sections were then incubated for 4 h in avidin conjugated to Pacific Blue (Invitrogen; 1:1000), rinsed, mounted in anti-fade medium and stored at −20 °C.

Thirty two retrogradely labelled NK1r-immunoreactive lamina I neurons were selected from the contralateral (right) dorsal horn of these animals: 14 from sections reacted to reveal NK1r/Fluorogold/GluA2/GluA4 and 18 from sections reacted for NK1r/Fluorogold/GluA2/GluA1. Between 1 and 6 cells were obtained from each rat. The selected cells were scanned with a Bio-Rad Radiance 2100 confocal microscope with Argon, green HeNe and red diode lasers. Scans were carried out sequentially (to avoid fluorescent bleedthrough) with a 60× oil-immersion lens and a z-step of 0.3 or 1 μm, and these were analysed with Neurolucida for Confocal software (MicroBrightField, Colchester, VT, USA). The scans acquired with the 1 μm z-step were used to reconstruct the cell bodies and dendritic trees of the neurons, and the maximum cross-sectional area of the soma was determined for each cell (Puskár et al., 2001; Al Ghamdi et al., 2009). Synaptic puncta on the dendrites of the cells were examined in scans acquired with a z-step of 0.3 μm. Because the penetration of immunostaining for synaptic AMPAr subunits is restricted (Todd et al., 2009), only cells with dendrites that approached the section surface were selected, and only those regions of dendrite that lay close to this surface were analysed. Cells were included in the sample if they had at least 100 μm length of dendrite that showed punctate AMPAr staining, or if more than 20 AMPAr puncta could be identified on their dendrites. For each cell, the somatodendritic morphology was examined and the cell was assigned to multipolar, pyramidal or fusiform classes, or was categorized as unclassified, according to standard criteria (Zhang et al., 1996; Zhang and Craig, 1997; Spike et al., 2003).

We have provided evidence that virtually all AMPAr-containing synapses in the spinal cord of unstimulated rats have GluA2 subunits, which coexist with various combinations of GluA1, GluA3 and GluA4 (Nagy et al., 2004a; Polgár et al., 2008b). Since GluA2-lacking AMPArs are Ca^2+^-permeable (Burnashev et al., 1992), this finding indicates that synapses with Ca^2+^-permeable AMPArs will also possess GluA2-containing (Ca^2+^-impermeable) receptors. The GluA2 antibody was therefore used to identify AMPAr-containing synapses in this study. Confocal z-series were initially viewed such that only the NK1r and GluA2 immunostaining were visible, and the locations of all of the GluA2-immunoreactive puncta that were in the membrane of the selected dendritic regions of these cells were identified and plotted onto the reconstructions. The files representing the other subunit (GluA4 or GluA1) were then viewed, and the presence or absence of staining for these subunits was determined for each GluA2 punctum. For certain cells, the lengths of all of the AMPAr puncta that were identified on their dendrites were measured (Todd et al., 2009). The lengths of the dendritic regions that were analysed for each cell were also measured, and from this the density of puncta per 100 μm was calculated. Dendritic length (rather than surface area) was used in this analysis, because puncta in the dendritic membrane could not be identified in regions of the membrane that were oblique or parallel to the section surface. The lengths of synaptic puncta on the projection neurons were compared with a population of 300 GluA2-immunoreactive puncta in lamina I that were not associated with NK1r-immunoreactive profiles. These were selected by placing a 5×5 μm^2^ grid over confocal scans from three rats and selecting puncta that were closest to the bottom right corners of the grid squares. In this way, 100 puncta were chosen in sections from each of three rats.

### AMPArs at synapses involving lamina I projection neurons and peptidergic primary afferents

In order to look for evidence that GluA4-containing AMPArs were present at synapses formed by peptidergic afferents, lumbar spinal cord sections from three rats that had received Fluorogold injections into LPb were reacted by a similar method to that described above, except that guinea-pig antibody against calcitonin gene-related peptide (CGRP; Bachem, St. Helens, UK; 1:50,000) was used instead of anti-NK1r. Again, this was revealed with a TSA method, and sections were then pepsin-treated and incubated in primary antibodies against Fluorogold, GluA2 and GluA4. These were revealed with the same combination of fluorescent/biotinylated secondary antibodies and avidin–Pacific Blue.

We have previously reported that NK1r-immunoreactive lamina I projection neurons receive a large number of contacts from peptidergic primary afferents, which can be identified by the presence of CGRP (Ju et al., 1987; Naim et al., 1997; Todd, 2002). These cells have regions of somatodendritic membrane with a very high density of such contacts, while projection neurons in this lamina that are not NK1r-immunoreactive do not receive a dense input from peptidergic afferents (Todd, 2002; Polgár et al., 2008a). Sections were examined with the confocal microscope as described above, and four retrogradely labelled cells that had dendrites with numerous contacts from CGRP-immunoreactive boutons were identified (one or two cells from each experiment). Confocal microscopy was used to scan regions of the dendrites of these cells with many CGRP contacts that were sufficiently close to the section surface to have immunostaining for the AMPAr subunits. Scans were obtained through a 60× oil-immersion lens as described above. GluA2^+^/GluA4^+^ puncta on these regions of dendrite were identified and plotted onto reconstructions of the dendritic tree with Neurolucida for Confocal software (as described above). The lengths of the puncta were measured and the presence or absence of CGRP boutons in contact with each punctum was noted.

### Contacts between glutamatergic boutons and NK1r-immunoreactive projection neurons

Since the density of AMPAr puncta on dendrites of the large and small NK1r-expressing projection neurons was found to differ (see below), we examined the density of contacts that these cells received from glutamatergic axons, which were identified with antibodies against CGRP and the vesicular glutamate transporter 2 (VGLUT2). This combination of antibodies should reveal the great majority of glutamatergic axons in lamina I. In the rat, all peptidergic primary afferents (which provide the major unmyelinated afferent input to this lamina) are thought to contain CGRP (Ju et al., 1987), and over 80% of the lamina I boutons labelled by transganglionic transport of cholera toxin B subunit (which correspond to Aδ nociceptors) express VGLUT2 (Todd et al., 2003). Lamina I is also innervated by many excitatory interneurons with cell bodies in laminae I–II (Gobel, 1978; Grudt and Perl, 2002; Lu and Perl, 2005; Cordero-Erausquin et al., 2009) that express VGLUT2 (Todd et al., 2003; Maxwell et al., 2007) and are likely to be the major source of VGLUT2-immunoreactive boutons in this lamina. Very few VGLUT1-containing axons are present in lamina I (Todd et al., 2003), and although Seal et al. (2009) have identified a plexus of VGLUT3-containing axons (corresponding to low-threshold unmyelinated primary afferents) in laminae I and II in the mouse, we have found that this plexus is largely restricted to lamina II in the rat (A.J. Todd and E. Polgár, unpublished observations).

Horizontal sections from four rats that had received retrograde injections of Fluorogold into LPb were incubated for 3 days in rabbit anti-NK1r (1:10,000), guinea pig anti-VGLUT2 (Millipore; 1:5000) and sheep anti-CGRP (Enzo Life Sciences, Exeter, UK; 1:10,000), overnight in appropriate species-specific secondary antibodies raised in donkey and conjugated to Alexa 488 (Invitrogen, 1:500), Rhodamine Red or Cy5 (Jackson Immunoresearch; 1:100) and then mounted in anti-fade medium. Twenty-four NK1r-immunoreactive retrogradely labelled lamina I neurons were selected (five to seven from each rat) and scanned with the confocal microscope, as described above. The scans were used to reconstruct the cell bodies and dendrites with Neurolucida for Confocal, and the cross-sectional area of the soma of each cell was determined. Contacts from CGRP- or VGLUT2-immunoreactive boutons were plotted onto regions of the dendritic tree (at least 100 μm length of dendrite for each cell), which were selected from areas that showed very good penetration of immunostaining for both CGRP and VGLUT2. For each cell, the density of all contacts (i.e. those from boutons that were CGRP- and/or VGLUT2-immunoreactive) was determined, as well as the proportion of these that involved a CGRP-immunoreactive bouton.

### Responses of NK1r-immunoreactive lamina I neurons to noxious stimuli

While this work was being carried out we reported that NK1r-expressing projection neurons in lamina I could be distinguished from interneurons based on soma size (Al Ghamdi et al., 2009). Specifically, we showed that 99% of the cells with soma cross-sectional areas >200 μm^2^ are projection neurons, while the great majority (>95%) of those below this size are interneurons. This part of the study was therefore carried out on rats that had not received injections of retrograde tracer and soma size was used to identify NK1r-immunoreactive cells as putative projection neurons or interneurons. Nine rats were anaesthetized with urethane (1.3 g/kg ip) and received one of the following noxious stimuli: (1) injection of 250 μg capsaicin (Sigma, Poole, UK; dissolved in 25 μl of 7% Tween 80 in saline) into the plantar surface of the left hindpaw (*n*=3), (2) pinching of folds of skin at 12 points on the left hindpaw (six each on the dorsal and ventral surfaces, applied for 5 s at each point; *n*=3), (3) immersion of the left hindpaw in water at 52 °C for 45 s (*n*=3). The animals remained anaesthetized for 5 min after the end of the stimulus, and were then perfused through the heart with 4% formaldehyde.

The L4 and L5 segments were removed and notched on the right side (to allow orientation), post-fixed overnight and cut into 60 μm thick horizontal sections with a vibrating microtome. These were reacted for 3 days in rabbit anti-NK1r (1:10,000) and mouse monoclonal antibody against phosphorylated extracellular signal-regulated kinases (pERK; Cell Signalling, Beverley, MA, USA; 1:1000), incubated overnight in appropriate species-specific secondary antibodies raised in donkey and conjugated to Alexa 488 (Invitrogen; 1:500) or Cy5 (Jackson Immunoresearch; 1:100), and then mounted in antifade medium and stored at −20 °C.

From the rats that had received capsaicin or the pinch stimulus, sections from both segments were initially scanned through the confocal microscope at low magnification, and the regions of lamina I that contained numerous pERK-immunoreactive cells were identified. These regions were then scanned through a 40× oil-immersion lens to produce overlapping fields of image stacks, with a z-separation of 2 μm. The files corresponding to NK1r immunostaining were analysed with Neurolucida for Confocal software. NK1r-immunoreactive cells were selected, and their cell bodies and proximal dendrites were drawn. Cross-sectional areas of cell bodies were measured. The files containing pERK immunostaining were then viewed and the presence or absence of pERK in each of the selected cells was determined. Tissue from the rats that received noxious thermal stimulation was scanned and analysed in the same way, except that only the L4 segment was examined.

NK1r-immunoreactive lamina I neurons with soma areas >200 μm^2^ were assumed to be projection neurons (Al Ghamdi et al., 2009), and were divided into two groups: those with somata >300 μm^2^ and those with somata of 200–300 μm^2^. Only ∼10% of NK1r-positive projection neurons have somata smaller than 200 μm^2^, and these are greatly outnumbered by small NK1r-immunoreactive interneurons, which would be expected to make up the vast majority of the cells in this size range (Al Ghamdi et al., 2009).

### Characterisation of antibodies

The rabbit NK1r antibody was raised against a peptide corresponding to amino acids 393-407 of the rat receptor conjugated to keyhole limpet haemocyanin, and staining with this antibody is absent in the brains of NK1r knock-out mice (Ptak et al., 2002). The two CGRP antibodies detect both α and β forms of the peptide. The VGLUT2 antibody was raised against a synthetic peptide from the rat VGLUT2 and we have shown that it stains identical structures to a well-characterized rabbit antibody against the transporter (Todd et al., 2003). The GluR1, GluR2 and GluR4 antibodies are all well-characterized and we have shown that staining with each of them is absent in sections from corresponding knock-out mice which have been processed with the pepsin antigen-retrieval method (Polgár et al., 2008b; Todd et al., 2009). The monoclonal pERK antibody detects both ERK1 and ERK2 that are dually phosphorylated at Thr202 and Tyr204 sites, and does not cross-react with JNK or p38 MAP kinase that are phosphorylated at the corresponding residues (manufacturer's specification).

## Results

### Injection sites

All Fluorogold injections resulted in extensive filling of the left LPb, with variable spread into surrounding regions, including the medial parabrachial area and the Kölliker-Fuse and cuneiform nuclei. Six of these injection sites have been illustrated previously (experiment 2 of Al Ghamdi et al., 2009; experiments 5–8 and 10 of Polgár et al., 2010).

### AMPAr puncta on NK1r-immunoreactive lamina I projection neurons

The distribution of punctate staining for GluA1, GluA2 and GluA4 in lamina I was similar to that described previously in studies that have used antigen retrieval with pepsin (Nagy et al., 2004a; Polgár et al., 2008a,b). Numerous GluA2 puncta were distributed throughout the neuropil and many of these were also GluA1-immunoreactive. In sections reacted with anti-GluA4, scattered puncta that were positive for both GluA2 and GluA4 were present, and these typically occurred in clusters that appeared to outline dendritic shafts. Although we did not analyse the relationship between AMPAr subunits in detail, we observed that the vast majority of puncta immunoreactive with either GluA1 or GluA4 antibodies were also GluA2-positive, as reported previously (Polgár et al., 2008b). Numerous Fluorogold-labelled cells were seen in lamina I on the contralateral (right) side following injections into the LPb, and most of these were NK1r-immunoreactive.

**Fig. 1:**
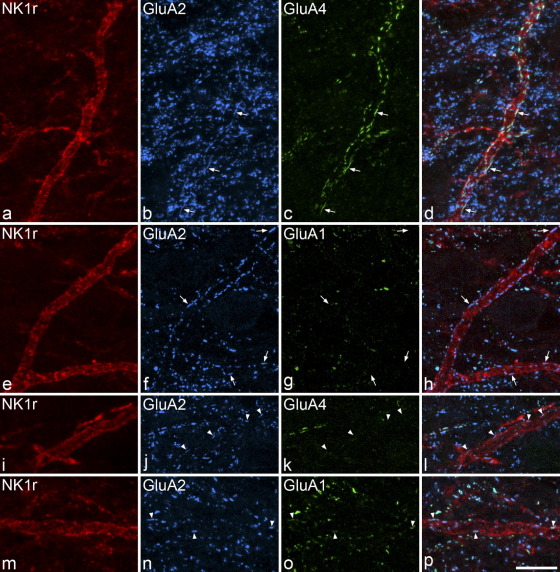
Confocal images showing AMPAr subunit immunoreactivity on the dendrites of large and small NK1r-expressing projection neurons in horizontal sections through lamina I. All images are from horizontal sections that had undergone a TSA reaction to reveal NK1r (red) followed by antigen retrieval with pepsin and immunostaining for GluA2 (blue) and either GluA4 or GluA1 (green). (a–d) Part of a dendrite belonging to a large retrogradely labelled cell (soma area 488 μm^2^) is outlined by NK1r immunostaining and is associated with numerous GluA2-immunoreactive puncta (some indicated with arrows) that are often elongated and that are also stained with the GluA4 antibody. Many GluA2^+^/GluA4^−^ puncta are seen in the surrounding neuropil and these are generally smaller than the puncta on the dendrite. (e–h) Part of the dendrite of another large NK1r^+^ projection cell (soma area 532 μm^2^) is associated with GluA2^+^ puncta that are not GluA1-immunoreactive (some of these are indicated with arrows). (i–l) The dendrite of a small NK1r^+^ projection neuron (soma area 211 μm^2^) has GluA2^+^ puncta (arrowheads) that are not GluA4-immunoreactive. (m–p) A dendrite belonging to another small NK1r^+^ projection cell (soma area 228 μm^2^) has GluA2^+^ puncta that are also GluA1-immunoreactive (arrowheads). Note that the density of GluA2 puncta is much lower on the dendrites of the small cells than on the dendrites of the large cells. Although there are several GluA2^+^ puncta close to the NK1r-immunoreactive dendrite in (m–p), these are not actually in the membrane. The images are projections of 11 (a–d), 10 (e–h), 3 (i–l) or 6 (m–p) optical sections at 0.3 μm z-separation. Scale bar=10 μm.

All of the selected projection neurons had GluA2 puncta that were judged to be in their dendritic membranes, based on the alignment of these puncta with the NK1r-immunostaining that outlined their dendrites (Fig. 1). Altogether, 2033 puncta were identified on the 32 cells examined (9–199 per cell). During the course of this analysis, it became apparent that NK1r-immunoreactive projection neurons differed in their pattern of AMPAr subunit expression (Table 1). Fourteen of the cells were from sections that had been reacted with GluA4, and nine of these had GluA2 puncta that were GluA4-positive (Fig. 1a–d), while five had GluA2 puncta that were GluaA4-negative (Fig. 1i–l). The remaining 18 cells were from sections reacted with GluA1: 13 of these had GluA2 puncta that were negative for GluA1 (Fig. 1e–h), while five had GluA2 puncta that were GluA1-positive (Fig. 1m–p). For each cell, there was a high degree of consistency in AMPAr immunostaining (Table 1): for the nine cells with GluA4-positive puncta 97%–100% (mean 99%) of GluA2 puncta were GluA4-immunoreactive, while for the five cells with GluA1-positive puncta the proportions of GluA2 puncta that were GluA1-immunoreactive were 92%–100% (mean 98%).

**Fig. 2:**
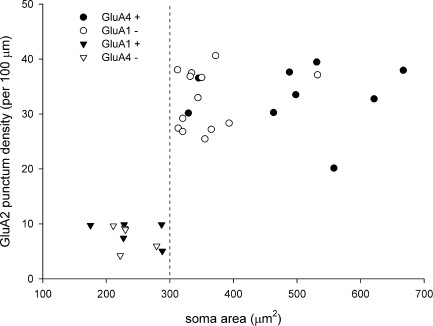
Scatter plot showing the relation of soma area to the density of GluA2 puncta on the dendrites for the 32 NK1r-expressing projection neurons that were analysed. Cells were also tested for the presence of either GluA4 or GluA1 and symbols indicate the presence or absence of immunostaining for these subunits at their GluA2 puncta. The dashed line corresponds to a soma area of 300 μm^2^.

The sizes of cells in these two groups were found to differ significantly. The soma areas of the cells for which GluA2 puncta were either GluA4-positive or GluA1-negative (312–667 μm^2^, median 360 μm^2^, *n*=22) were all larger than those of cells with GluA2 puncta that were GluA4-negative or GluA1-positive (175–288 μm^2^, median 229 μm^2^, *n*=10; Table 1, Fig. 2). This difference was highly significant (*P*<0.0001, Mann–Whitney *U*-test).

We found that the dendrites of the large (GluA4^+^/GluA1^**−**^) projection cells had many AMPAr puncta, which often outlined them. However, puncta were far less numerous on the dendrites of the smaller (GluA4^**−**^/GluA1^+^) cells (Fig. 1). We therefore measured the density of puncta on these two populations (Fig. 2). The mean density of puncta per 100 μm of dendrite on the large cells was 32.9±5.5 SD (*n*=22 cells), while that on the smaller cells was 8±2.2 (*n*=10 cells). The difference between these values was highly significant (*P*<0.001; *t*-test). Although there was a relation between soma size and density of dendritic AMPAr puncta across the whole population, there was no correlation between these values within each group (large cells *r*=0.11, Pearson correlation coefficient, *P*=0.61; small cells *r*=−0.37, *P*=0.29). To test whether the density of puncta was influenced by distance from the soma, we compared the density for dendrites less than 150 μm from the soma (proximal) with those greater than 150 μm from the soma (distal) for the 22 large cells. The mean density per 100 μm on proximal dendrites was 33.2±9 SD (*n*=21 cells), while that on distal dendrites was 30.9±7 SD (*n*=12 cells), and these values were not significantly different (*P*=0.45, *t*-test).

**Fig. 3:**
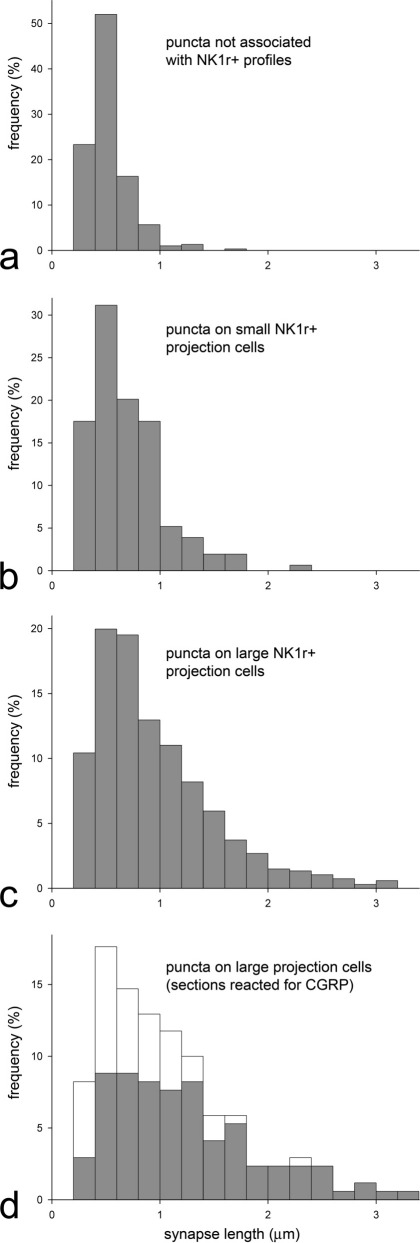
Sizes of AMPAr-immunoreactive puncta on NK1r-expressing lamina I projection neurons compared to those not associated with these cells. The histograms show the frequency distribution of puncta of different sizes for: (a) those that were not associated with NK1r^+^ profiles (*n*=300 puncta), (b) those present on the dendrites of small projection cells (soma area <300 μm^2^, *n*=154 puncta on 10 cells) and (c) those on large projection cells (soma area >300 μm^2^, *n*=671 puncta on nine cells). (d) shows the frequency distribution of puncta associated with the four projection neurons that were analysed in sections reacted for CGRP, GluA2 and GluA4. The grey portions of each bar represent puncta that were apposed to CGRP-immunoreactive boutons, while the white parts correspond to puncta not associated with these boutons. For further details, see text.

We found that many of the AMPAr puncta on the large lamina I projection neurons were very elongated (Fig. 1a–h), and we therefore compared lengths of puncta on the two populations of projection neurons (large and small), with GluA2-immunoreactive puncta in lamina I that were not associated with NK1r profiles (Fig. 3). The lengths of puncta were measured on the nine large cells that were tested for GluA4 and these varied from 0.3 to 3.2 μm (median 0.9; *n*=671). Since puncta were less numerous on the smaller cells, we measured their lengths on all 10 cells in this group and found that these varied from 0.3 to 2.3 μm (median 0.7; *n*=154). The lengths of the GluA2 puncta that were not associated with NK1r-immunoreactive profiles varied from 0.3 to 1.7 μm (median 0.5; *n*=300). The differences between these three populations were highly significant (Kruskall Wallis, *P*<0.0001). Post-hoc Mann–Whitney U-tests with sequential Bonferroni correction showed a highly significant difference between each of the three groups (*P*<0.001). For the nine large cells for which punctum size was analysed we compared lengths of puncta on proximal (<150 μm from soma) and distal (>150 μm from soma) dendrites. Although there was considerable overlap between the two groups (ranges 0.3–3.2 μm, *n*=482 for those on proximal dendrites, 0.3–3.2 μm, *n*=189 for those on distal dendrites) the median value for distal puncta (1.1 μm) was significantly larger than that for proximal puncta (0.8 μm) (Mann–Whitney *U*-test, *P*<0.0001), indicating that glutamatergic synapses tended to be larger on distal dendrites.

### Association with CGRP-immunoreactive boutons

**Fig. 4:**
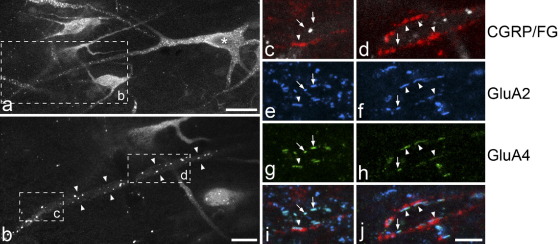
Confocal images showing the relation of CGRP-immunoreactive boutons to GluA2^+^/GluA4^+^ puncta on the dendrites of a retrogradely-labelled lamina I neuron. (a) A low magnification image reveals several Fluorogold (FG) labelled cells in a horizontal section through lamina I. One of these is identified with an asterisk. The box indicates the region shown at higher magnification in (b). (b) Part of the dendrite of the cell marked with the asterisk is seen (between arrowheads) and contains several bright granules of FG. Boxes represent areas shown in the subsequent images. (c, e, g, i) and (d, f, h, j) show two regions of the dendrite of this cell which have been scanned to reveal Fluorogold (white), CGRP (red), GluA2 (blue) and GluA4 (green). Both these regions have several GluA2^+^/GluA4^+^ puncta. Some of these (arrowheads) are apposed to CGRP-immunoreactive boutons, while others (arrows) are not. The images are projections of seven optical sections at 1 μm z-separation (a) and of five (b), three (c, e, g, i) or two (d, f, h, j) optical sections at 0.5 μm separation. Scale bars=25 μm (a), 10 μm (b), 5 μm (c–j).

Frequency histograms showed that although the majority of puncta on the large projection neurons were <1 μm long, these cells had a significant population of much larger puncta (Fig. 3). We therefore tested whether these large puncta were associated with peptidergic primary afferents, which provide a powerful synaptic input to these cells (Todd et al., 2002). All four of the cells examined in this part of the study had somata larger than 300 μm^2^ (340–430 μm^2^) and a high density of contacts from CGRP-immunoreactive boutons. All of the cells had GluA2^+^/GluA4^+^ puncta and many, but not all of these were adjacent to CGRP-immunoreactive boutons (Fig. 4). Altogether, 170 GluA2^+^/GluA4^+^ puncta were found on these cells (16–65 per cell): 113 of these were associated with CGRP-positive boutons, while the remaining 57 were not. The median length of CGRP-associated puncta was 1.2 μm (range 0.3–3.3 μm), while that for the remaining puncta was 0.7 μm (range 0.4–2.4 μm), and this difference was highly significant (*P*<0.0001, Mann–Whitney U-test). Comparison of the lengths of these puncta with those seen on the nine GluA4-expressing large cells in the first part of the study showed a similar overall frequency distribution (Fig. 3c, d). However, while puncta that were associated with CGRP varicosities included both small and large ones, the remainder were seldom >1.8 μm.

### Contacts from VGLUT2 and CGRP boutons

**Fig. 5:**
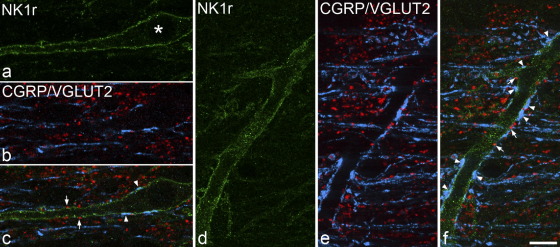
Confocal images showing contacts from CGRP- and VGLUT2-containing boutons onto the dendrites of NK1r-expressing lamina I projection neurons. (a) shows the soma (asterisk) and part of a dendrite belonging to a small (soma area=276 μm^2^) projection neuron, which are outlined with the NK1r (green). (b) The corresponding field scanned to reveal CGRP (blue) and VGLUT2 (red). (c) The merged image shows scattered contacts from CGRP- and VGLUT2-immunoreactive boutons (some of which are indicated with arrowheads and arrows, respectively). (d) Part of the dendrite of a large projection neuron (soma area 498 μm^2^) is outlined by the NK1r. (e) This field scanned to reveal CGRP and VGLUT2. (f) The merged image shows numerous contacts from CGRP- and VGLUT2-immunoreactive boutons (some of which are indicated with arrowheads and arrows, respectively). The images are projections of two (a–c) and three (d–f) optical sections at 0.5 μm separation. Scale bar=10 μm.

**Fig. 6:**
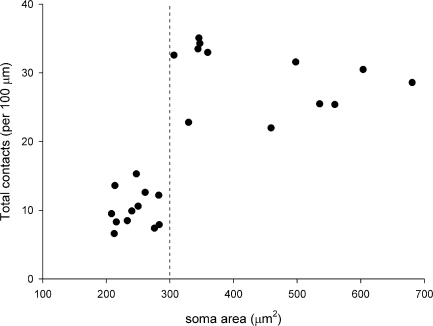
Scatter plot showing the relation of soma area to the density of contacts from boutons that were CGRP- and/or VGLUT2-immunoreactive on the dendrites for the 24 NK1r-expressing projection neurons that were analysed. The dashed line corresponds to a soma area of 300 μm^2^.

Lamina I contained numerous CGRP- and VGLUT2-immunoreactive boutons. As reported previously (Todd et al., 2003), some CGRP-positive boutons in this lamina showed weak VGLUT2 immunoreactivity, while others did not have detectable immunostaining. All of the 24 cells examined in this part of the study received contacts from boutons with CGRP- and/or VGLUT2-immunoreactivity (Fig. 5). However, the contact density varied considerably between cells, and was related to size of the soma (Fig. 6). A scatter plot of soma size against density of contacts from all CGRP- and/or VGLUT2-immunoreactive boutons revealed that there were two distinct groups of cells: those with a soma area >300 μm^2^ had mean contact densities of 29.6 (±4.6 SD) per 100 μm (*n*=12), while for those with soma areas <300 μm^2^ the mean was 10.2 (±2.7) per 100 μm (*n*=12). This difference was highly significant (*P*<0.0001, *t*-test). Within each group there was no correlation between soma size and contact density (large cells *r*=−0.36, *P*=0.26; small cells *r*=0.08, *P*=0.8). The proportion of these boutons that were CGRP-immunoreactive did not differ significantly between the two groups (42%–76%, mean 56% for the large cells; 20%–88%, mean 50% for the small cells; *P*=0.35, *t*-test). The mean density of contacts from CGRP-immunoreactive varicosities was 16.5 (±3.8) per 100 μm for the large cells and 4.9 (±1.7)/100 μm for the small cells.

Since large AMPAr puncta (which were often associated with CGRP-immunoreactive varicosities) were somewhat more common on distal dendrites of the large cells (see above), we examined whether this could result from a differential distribution of excitatory inputs. We found that percentages of contacts that involved a CGRP-positive bouton were 57.2 for proximal and 57.5 for distal dendrites, and these values did not differ significantly (data pooled from 12 cells, *P*=0.79, Chi-squared-test). This indicates that the higher frequency of larger puncta on distal dendrites of these cells is not accounted for by a differential distribution of peptidergic primary afferent input.

### Morphology

Analysis of both AMPAr-immunostaining and density of contacts from glutamatergic boutons indicated that there are two distinct populations of NK1r-expressing projection neurons in lamina I. To investigate the morphology of cells in these two groups, we pooled data from the 32 cells examined in the first part of the study with the 24 for which contacts were analysed. These were then divided into two groups: large (GluA4^+^, GluA1^**−**^ or with a high density of contacts, >20/100 μm length of dendrite, *n*=34) and small (GluA4^**−**^, GluA1^+^ or with a low density of contacts <20/100 μm length of dendrite, *n*=22) cells. The large group contained 10 pyramidal, 12 multipolar and 12 fusiform cells, while the small group consisted of four pyramidal cells, five multipolar, 12 fusiform and one unclassified cell. Although fusiform cells were somewhat over-represented among the small population, these differences were not significant (*P*=0.23, Fisher's exact Probability test).

### pERK in NK1r-immunoreactive lamina I neurons

**Fig. 7:**
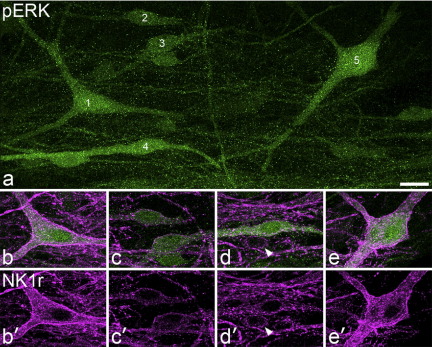
Confocal images showing pERK-immunoreactivity in NK1r-expressing lamina I neurons following injection of capsaicin into the hindpaw. (a) A projected confocal image stack from a horizontal section through the ipsilateral side of the L5 segment. Several pERK-immunoreactive neurons can be seen and five of these are numbered. (b–e, b′–e′) show more limited projections through cells 1 (b, b′), 2, 3 (c, c′), 4 (d, d′) and 5 (e, e′). Each of these cells is immunostained for both NK1r and pERK, and there is internalization of the receptor. Cells 1, 3 and 5 had somata >300 μm^2^ cross-sectional area (519, 309, 614 μm^2^, respectively), while cells 2 and 4 were in the 200–300 μm^2^ range (203, 246 μm^2^, respectively). A small NK1r-immunoreactive cell (soma area 149 μm^2^) that was negative for pERK is marked with an arrowhead in (d). Images are projections of nine (a), four (b, e) or three (c, d) optical sections at 2 μm z-spacing. Scale bar=20 μm.

Quantitative results from the rats that had received capsaicin or the pinch stimulus are shown in Table 2, and examples of pERK and NK1r immunostaining are illustrated in Fig. 7. Numerous pERK-positive cells were seen in lamina I in the medial part of the dorsal horn on the left side (ipsilateral to the stimulus) in both L4 and L5 segments, while very few were present on the contralateral side. Most of the NK1r-immunoreactive cells with soma cross-sectional areas >200 μm^2^ were pERK-positive following capsaicin treatment. When data from the three rats were pooled, pERK was found in 83% of the NK1r-positive cells >300 μm^2^ and 77% of those between 200 and 300 μm^2^. However, only 39% of the cells smaller than 200 μm^2^ were pERK-positive. In the three rats that had received the pinch stimulus, 75% of NK1r-positive cells with cell bodies >300 μm^2^, 54% of those between 200 and 300 μm^2^, and 28% of those <200 μm^2^ contained pERK. One way ANOVA with Tukey test post hoc revealed that in the capsacin-treated group the expression of pERK differed between the cells with somata <200 μm^2^ and both other groups (*P*<0.001), but not between the 200–300 μm^2^ and >300 μm^2^ groups. In contrast all three size groups differed significantly from each other in pERK expression in the animals that had received the pinch stimulus (*P*<0.05). In the L4 segments of the rats stimulated with noxious heat all of the 59 NK1r-immunoreactive cells >300 μm^2^ (17–22 per experiment) and all of the 47 cells between 200 and 300 μm^2^ (15–16 per experiment) were pERK-positive, as were 70% of the cells <200 μm^2^ (135 cells, 49–80 per experiment). We therefore did not analyse the L5 segments in these animals. With each type of noxious stimulus, many of the pERK-positive NK1r-immunoreactive cells showed internalization of the receptor (Fig. 7).

## Discussion

The main finding of this study is that there are two distinct populations of NK1r-expressing projection neurons in lamina I that differ in terms of excitatory input: large cells have AMPArs that contain GluA4 but not GluA1, and a high density of glutamatergic synapses, while small cells have AMPArs with GluA1 but not GluA4, and a significantly lower synaptic density. Both types respond to noxious stimulation, although fewer of the small cells were pERK-positive in response to pinch.

### Two populations of NK1r-expressing lamina I projection neurons

NK1rs are expressed by the great majority of lamina I projection neurons in the rat, and given the functional diversity of these cells (Bester et al., 2000; Andrew, 2009) it is unlikely that expression of the receptor defines a homogeneous population. However, previous attempts to classify lamina I projection neurons on purely morphological grounds have proved controversial (Han et al., 1998; Todd et al., 2002; Spike et al., 2003; Almarestani et al., 2007; Al-Khater and Todd, 2009). The present results provide an alternative method for classifying the NK1r-expressing cells. Al Ghamdi et al. (2009) reported that 49% of NK1r-immunoreactive lamina I projection neurons had soma areas >300 μm^2^, and although there will presumably be overlap between soma sizes of the two types of projection cell identified in the present study, this observation suggests that they will occur with approximately equal frequency. However, lumbar NK1r-expressing spinothalamic lamina I neurons (which also project to the parabrachial area) are significantly larger than other NK1r-positive spinoparabrachial cells in this lamina (Al-Khater and Todd, 2009), so the large, densely innervated neurons with GluA4-containing AMPArs are likely to be over-represented within the spinothalamic population.

Consistent with findings of physiological studies, which have shown that virtually all spinoparabrachial lamina I neurons in the rat are excited by noxious stimuli (Bester et al., 2000; Andrew, 2009), our results suggest that all of the cells in both populations respond to noxious heat, with the great majority also being activated by capsaicin. The difference in the proportion that responded to pinch could result from differences in receptive field sizes between the two types. If projection neurons with larger somata have more extensive dendritic trees, this may be reflected in larger peripheral receptive fields, due to the somatotopic organization of primary afferent input to lamina I. If this is the case, there would be a higher probability that their receptive fields extended into skin regions that had been stimulated in these experiments. Therefore the two classes of NK1r-expressing projection cell may not differ in the types of noxious stimulus to which they respond. With each type of stimulus fewer NK1r-immunoreactive cells with somata <200 μm^2^ were pERK-positive, and it is therefore possible that fewer interneurons with the receptor are activated under these conditions. Nonetheless, results from the heat experiments indicate that most of these cells are also activated by noxious stimuli.

### Glutamatergic inputs

We have previously reported that NK1r-immunoreactive lamina I projection neurons received numerous contacts from substance P-containing primary afferents, which were identified by the presence of both substance P and CGRP (Todd et al., 2002). The cells analysed in that study were probably of the large type, as they all had regions of dendrite with a high density of contacts from peptidergic afferents, a feature that was only seen on the large cells in this study. It is likely that the great majority of CGRP-immunoreactive boutons that contacted the cells in the present study belonged to substance P-containing primary afferents, since most CGRP-immunoreactive boutons in lamina I are also substance P-immunoreactive, and the density of substance P/CGRP contacts on dendrites that we observed in our previous study (16.7 per 100 μm) (Todd et al., 2002) was very similar to the mean density of CGRP contacts seen here on the large cells (16.5 per 100 μm).

Although we cannot identify the sources of individual VGLUT2^+^/CGRP^−^ boutons that contact these cells, they presumably include Aδ nociceptors, which provide monosynaptic input to at least some lamina I projection neurons in the rat (Andrew, 2010). It is unlikely that many belong to non-peptidergic C fibres since most of these, which can be identified by binding of the lectin IB4 (Todd, 2002), terminate in lamina II. Dhaka et al. (2008) described a population of thermoreceptive C fibres that express TRPM8, lack CGRP and terminate in lamina I, but these did not appear to make contact with NK1r-immunoreactive neurons. The majority of the VGLUT2^+^/CGRP^−^ boutons are therefore likely to originate from local excitatory interneurons (Lu and Perl, 2005), which convey various types of sensory information, including that originating from myelinated and unmyelinated low-threshold mechanoreceptive afferents. These inputs underlie the tactile responses that can be detected in some projection cells (Bester et al., 2000; Andrew, 2010), and may contribute to allodynia in pathological pain states (Torsney and MacDermott, 2006). Differences between the two populations in the density of contacts from VGLUT2-immunoreactive boutons may be reflected in differences in the proportions with monosynaptic Aδ input and/or wide dynamic range receptive fields.

Our results suggest that at least half of the excitatory synaptic input to both large and small NK1r-expressing projection neurons in lamina I is from peptidergic primary afferent nociceptors. This contrasts with the excitatory inputs to another population of lamina I projection neurons (large cells that lack the NK1r and are coated with gephyrin puncta), which are densely innervated by VGLUT2-immunoreactive boutons, but receive few contacts from peptidergic afferents or Aδ nociceptors (Polgár et al., 2008a). These cells also respond to noxious stimulation (Puskár et al., 2001), but presumably receive much of this input via excitatory interneurons (Polgár et al., 2008a).

### AMPArs at synapses on projection neurons

Some of the AMPAr puncta seen on the large NK1r-immunoreactive projection neurons in this study were very elongated, and these were associated with CGRP-immunoreactive (primary afferent) boutons. These presumably represent large synapses containing many receptor molecules, and are therefore likely to generate large excitatory postsynaptic currents (EPSCs). This is consistent with the finding by Tong and MacDermott (2006) that primary afferent-evoked EPSCs on NK1r-expressing lamina I neurons were larger than those seen on non-NK1r cells in this lamina. The NK1r-positive cells recorded by Tong and MacDermott had larger capacitances than the non-NK1r cells (corresponding to larger soma sizes), and this group is therefore likely to have consisted mainly of projection neurons (Al Ghamdi et al., 2009). It has been suggested that synaptic size is correlated with release probability at excitatory synapses (Schikorski and Stevens, 1997), and the large synapses that we observed are therefore also likely to have a relatively high release probability. Since 56% of the glutamatergic contacts on the large cells were from CGRP-immunoreactive boutons and some of these were associated with the largest puncta, peptidergic primary afferents presumably provide the major excitatory input to these cells. We previously reported that there were very elongated AMPAr puncta on the dendrites of lamina III/IV NK1r-expressing projection neurons (Todd et al., 2009), and it is likely that these also correspond to synapses from peptidergic primary afferents, which densely innervate these cells (Naim et al., 1997). We also found that the AMPAr puncta on distal dendrites of the large lamina I projection neurons were significantly larger than those on proximal dendrites. This presumably means that more distal synapses would generate larger EPSCs, thus compensating for the greater attenuation that results from their increased distance from the soma (Magee and Cook, 2000).

All four AMPAr subunits are expressed by dorsal horn neurons (Furuyama et al., 1993; Henley et al., 1993; Tölle et al., 1993; Jakowec et al., 1995a,b; Spike et al., 1998; Engelman et al., 1999; Hartmann et al., 2004), but with a distinctive laminar distribution (Nagy et al., 2004a; Polgár et al., 2008b). In particular, GluA4-containing receptors are relatively uncommon in the superficial dorsal horn, being found at only 23% of glutamatergic synapses in lamina I and in <10% of those in lamina II. In contrast, over 60% of AMPAr puncta in this region contain GluA1 (Polgár et al., 2008b). Since the great majority of glutamatergic synapses in lamina I contain one or other of these subunits it is likely that the large NK1r-expressing projection neurons that were found to be GluA1-negative would have expressed GluA4, while the small GluA4-negative ones would have expressed GluA1. Since ∼80% of GluA1-negative and ∼60% of GluA1-positive AMPAr puncta in lamina I are GluA3-immunoreactive (Polgár et al., 2008b), it is likely that GluA3 will also be expressed by many NK1r-immunoreactive projection neurons in this lamina.

We have previously demonstrated that GluA4-containing AMPArs are present at synapses on two other types of dorsal horn projection neuron: the large gephyrin-coated lamina I cells (Polgár et al., 2008a) and NK1r-expressing neurons with somata in laminae III or IV and dorsal dendrites that extend into lamina I (Todd et al., 2009). Like the large lamina I NK1r-immunoreactive projection neurons seen in this study, both of these cell types were also found to have a very high density of glutamatergic synapses on their dendrites. These findings, taken together with the laminar distribution of AMPAr subunits, suggest that GluA4 is mainly or exclusively expressed by projection neurons in the superficial dorsal horn, with GluA1 being present at synapses on interneurons and on small projection neurons in lamina I. If this interpretation is correct, then at least a quarter of the glutamatergic synapses in lamina I would be associated with dendrites and cell bodies of projection neurons, even though these cells make up only ∼5% of the neuronal population in this lamina (Spike et al., 2003).

Ikeda et al. (2003, 2006) described two forms of LTP in NK1r-expressing lamina I projection neurons, one evoked by high- and the other by low-frequency stimulation of C fibres. The present findings highlight the importance of GluA4-containing AMPArs in nociceptive processing and raise the possibility that differences in the type of LTP seen by Ikeda et al. may be related to differential expression of GluA4 or GluA1.

## Figures and Tables

**Table 1:** Immunostaining for GluA4 and GluA1 among NK1r-immunoreactive projection neurons in lamina I

Immunostaining for GluA1/GluA4	Number of cells	Mean soma area (μm^2^)	Mean number of GluA2 puncta examined	% of GluA2 puncta positive for GluA4	% of GluA2 puncta positive for GluA1
GluA4^+^	9	500 (329–667)	75 (21–153)	99 (97–100)	
GluA1^−^	13	357 (312–532)	93 (44–199)		0
GluA1^+^	5	241 (175–288)	15 (10–24)		98 (92–100)
GluA4^−^	5	234 (211–279)	15 (9–27)	0	

Numbers in parentheses are the ranges within each group.

**Table 2:** pERK expression in NK1r-immunoreactive lamina I neurons

Stimulus	Experiment	Soma cross-sectional area		
		>300 μm^2^	200–300 μm^2^	<200 μm^2^
Capsaicin	1	53/62 (85)	34/41 (83)	56/116 (48)
Capsaicin	2	60/73 (82)	32/44 (73)	50/136 (37)
Capsaicin	3	33/40 (83)	17/23 (74)	29/92 (32)
Capsaicin (sum)		146/175 (83)	83/108 (77)	135/344 (39)
Pinch	4	59/79 (75)	21/40 (53)	50/181 (28)
Pinch	5	51/77 (66)	22/40 (55)	36/122 (30)
Pinch	6	58/67 (87)	17/32 (53)	23/90 (26)
Pinch (sum)		168/223 (75)	60/112 (54)	109/393 (28)

Proportions (and percentages) of NK1r-immunoreactive lamina I neurons of different sizes that were pERK-positive after capsacin and pinch stimuli. Cells with soma area <200 μm^2^ are considered to be interneurons, while those >200 μm^2^ are presumed to be projection neurons, based on the results of Al Ghamdi et al. (2009). After capsaicin stimulation frequency of pERK-immunostaining in interneurons was significantly less than that in both groups of projection neurons (*P*<0.001, one way ANOVA with Tukey's test *post hoc*). In contrast, all three size groups differed significantly in frequency in animals that had received the pinch stimulus (*P*<0.05, one way ANOVA with Tukey's test *post hoc*).
